# Descriptive Study on Attentional and Executive Functioning Following CAR-T Cell Therapy in Paediatric Patients with Acute Lymphoblastic Leukaemia

**DOI:** 10.1192/j.eurpsy.2025.1231

**Published:** 2025-08-26

**Authors:** C. Campoy-Lacasa, V. Galán-Gómez, B. González-Martínez, A. Pérez-Martínez, E. Fernández-Jiménez

**Affiliations:** 1 Department of Psychiatry, Clinical Psychology and Mental Health; 2 Department of Pediatric Hemato-Oncology, La Paz University Hospital; 3 Department of Pediatrics, Universidad Autónoma de Madrid; 4 CIBERER-ISCIII. IdiPAZ-CNIO Pediatric Onco-Hematology Clinical Research Unit, Spanish National Cancer Research Center (CNIO); 5 Hospital La Paz Institute for Health Research (IdiPAZ), Madrid; 6 Faculty of Social Sciences and Communication, Universidad Europea de Madrid, Villaviciosa de Odón, Spain

## Abstract

**Introduction:**

Chimeric antigen receptor–engineered T-cell (CAR-T) therapy is a newly approved treatment that has shown high remission rates among patients with acute lymphoblastic leukaemia. However, its neuropsychological impact remains largely unknown. To our knowledge, this is the first study to examine the neurocognitive effects of CAR-T therapy in children.

**Objectives:**

To analyse attentional and executive functioning outcomes in paediatric patients with acute lymphoblastic leukaemia after CAR-T therapy.

**Methods:**

This study was conducted at the Child Neuropsychology Unit of La Paz University Hospital in Madrid, Spain. Thirteen paediatric patients aged 7–16 years (mean age = 12 years and 1 month; 75% male) were assessed. Neurocognitive assessments were performed at an average of 1 year and 4 months after CAR-T therapy (ranging from 3 months to 3 years and 4 months). Neurocognitive measures included the Symbol Digit Modalities Test (SDMT), the Omission score from the Continuous Performance Test Third Edition (CPT 3), the Test of Nonverbal Intelligence Fourth Edition (TONI-4), and the Verbal Fluency Test (FAS). Descriptive analyses were conducted, including median, minimum and maximum scores for age- and sex-adjusted z-scores, with significant impairment defined as z < -1.5.

**Results:**

Findings revealed that 58.3% of the children scored significantly below norms on non-verbal abstract reasoning (TONI-4: Median = -1.53; Min = -3.27; Max = 1.27), 53.9% displayed significant sustained attentional deficits (Omission CPT 3: Median = -3.1; Min = -4.0; Max = 0.70), and 41.7% showed relevant impairments in both processing speed (SDMT: Median = -1.5; Min = -5.7; Max = 1.9) and phonological verbal fluency (FAS: Median = -0.7; Min = -2.9; Max = 1.5).

**Conclusions:**

Paediatric acute lymphoblastic leukaemia survivors treated with CAR-T therapy show deficits in processing speed, sustained attention, abstract reasoning, and phonological verbal fluency. These findings underscore the need for both short- and long-term neuropsychological monitoring, and tailored interventions targeting attentional and executive functioning deficits in this population. Future studies should replicate these analyses by using larger sample sizes.

**Disclosure of Interest:**

None Declared

